# Seasonal Occurrence and Biodiversity of Insects in an Arid Ecosystem: An Ecological Study of the King Abdulaziz Royal Reserve, Saudi Arabia

**DOI:** 10.3390/biology14030254

**Published:** 2025-03-02

**Authors:** Abdulrahaman S. Alzahrani, Moutaman Ali Kehail, Sara A. Almannaa, Areej H. Alkhalifa, Abdulaziz M. Alqahtani, Mohammed H. Altalhi, Hussein H. Alkhamis, Abdullah M. Alowaifeer, Abdulwahed Fahad Alrefaei

**Affiliations:** 1 The King Abdulaziz Royal Reserve (KARR), Riyadh 12213, Saudi Arabia; a.alzahrani@karnr.gov.sa (A.S.A.); s.almannaa@karnr.gov.sa (S.A.A.); t.abdulaziz@karnr.gov.sa (A.M.A.); t.mohammed@karnr.gov.sa (M.H.A.); 2 Green Sustainability Company for Environmental Services (GSCES), Riyadh 13326, Saudi Arabia; motaman@gsces.sa (M.A.K.); hussein@gsces.sa (H.H.A.); 3 Department of Biology, College of Science, Princess Nourah Bint Abdulrahman University, Riyadh 11671, Saudi Arabia; ahalkhalifa@pnu.edu.sa; 4 Department of Zoology, College of Science, King Saud University, P.O. Box 2455, Riyadh 11451, Saudi Arabia

**Keywords:** biodiversity, occurrence, insects, King Abdulaziz Royal Nature Reserve (KARR), Saudi Arabia

## Abstract

Each living organism thrives best in a habitat that provides optimal conditions for flourishing, reproduction, and distribution within a certain area. This study aims to investigate the seasonal variation in insect biodiversity across different sites of the King Abdulaziz Royal Reserve (KARR), located between E 45.19–46.57 and N 25.15–27.41, with a focus on assessing biodiversity, density and seasonal variation using active and passive methods, over the period from January to November 2023. A total of 68 sites within the study area were randomly selected for trap placement. The trapped specimens were labeled and transferred to plastic bottles half filled with 70% ethanol and then taken to the laboratory for counting and identification. Identification was based on morphological characteristics and appropriate identification keys, with the assistance of entomological expertise, and a list of local species. Simpson’s diversity index (D) was also calculated. The results revealed that, out of 6320 trapped insects, species were identified across six orders: Blattodea (termites), represented by 2 families and 2 species; Coleoptera, comprising 12 families and 38 species, of which 11 belonged to the family Tenebrionidae; Hemiptera, comprising 7 families and 9 species, 3 of which belonged to the family Lygaeidae; Hymenoptera, comprising 5 families and 15 species, 9 of which were from Formicidae; Lepidoptera, comprising 2 families and 3 species; and Orthoptera, comprising 3 families and 7 species, 4 of which were from family Acrididae. Insect biodiversity and abundance were observed to be relatively low during the winter (January–March) and autumn (October–November) seasons, while relatively higher densities were recorded during spring (May) and summer (August–September).

## Simple Summary

This study investigates the seasonal variation in insect biodiversity at the King Abdulaziz Royal Reserve (KARR) from January to November 2023. The researchers used active and passive methods to assess biodiversity and insect density across 68 randomly selected sites. A total of 6320 insects from six orders were identified, including species from Blattodea, Coleoptera, Hemiptera, Hymenoptera, Lepidoptera, and Orthoptera. The results showed that insect biodiversity was relatively the lowest in winter and autumn, while density peaked in spring and summer.

## 1. Introduction

In 2018, it was estimated that approximately one million insect species had been described and identified, accounting for more than 50% of all eukaryotes. The predicted number of new species described each year is around 20.000. The estimated total number of insect species, including those not yet identified, is believed to be 5.5 million, representing about 80% of all eukaryotes. Insects are classified into 24 orders. In terms of the number of species, Coleoptera (beetles: 386.500), Lepidoptera (butterflies and moths: 157.338), Diptera (flies and mosquitoes: 155.477), Hymenoptera (ants, bees, and wasps: 116.861), Hemiptera (true bugs: 103.590), and Orthoptera (locusts, grasshoppers, and crickets 23.855) are the dominant orders, while Mantophasmatodea (15) is the least abundant [1].

It was reported that some insect populations are in decline in their abundance, biomass, and species richness in many studied areas, while other insect populations are not. Generally, each insect species is affected by changes in the environment in different ways compared to other species (depending on the success of adaptation to survive). The declines of insect abundance have been attributed to reasons such as habitat loss (urbanization), pesticide (insecticides and herbicides) use on crops, and invasive species, which compete with the indigenous species [2]. During 2017, about 66 insect species were recorded as extinction species [3] all around the world, and many other insect species were documented by IUCN (the International Union for Conservation of Nature). This includes 97 species of Odanata, 91 species of Orthoptera, 72 species of Coleoptera, 51 species of Lepidoptera, 18 species of Hymenoptera, and 7 species of Blattodea [4]. Nowadays, more than 166.000 species of both fauna and flora populations have been documented within IUCN red list [5].

A nature reserve is a protected ecological area, mainly for fauna and flora [6]. It is also known as wildlife refuge, wildlife sanctuary, or nature conservation area. These areas usually fall into IUCN in different categories in addition to the local laws [7]. The establishment of nature reserves is an ecological way to protect biodiversity. It is also considered an effective method to protect the ecological environment from human activities that disturb habitat quality [8]. The assessment of biotic and abiotic components will help for planning different conservation scenarios [9] in the context of systematic conservation to form a resilient ecological system [10].

The Kingdom of Saudi Arabia (KSA) occupies an area of about 2.2 million km^2^. This area is characterized by different ecological systems, but it is classified as arid and semiarid. There are various ecosystems including valley, hills, deserts (rocky and sandy), and coastal areas that provide the optimal conditions for different fauna and flora to flourish. The ecological system of KSA hosts about 78 species of mammals, 550 species of birds, 130 species of reptiles, 7 species of amphibians, and an undefined number of fish, arthropods, and other invertebrates [11]. In 2018, the King Abdulaziz Royal Nature Reserve (KARR) was established. It occupies an area of 28.345 km^2^ (about 13% of KSA total area). It represents all the ecosystem of the KSA, except the coastal one. The KARR is working within its ability, under laws and legislations set by IUCN organization, to conserve biomass and to assess biodiversity [12].

The evolution of soil arthropod diversity in desert areas has been studied using both active and passive trapping techniques [13]. Arid conditions are driven by plant size, plant morphological characters, soil fertilization, plant nutritional content, and prey–predator interactions [14].

In arid areas, climate change (e.g., the elevation of temperature) increases the frequency of droughts and fluctuates rainfall pattern and rates, which in turn directly affects living organisms such as insect and plant populations. The unstable and unpredicted weather conditions may alter the normal physiological process of all living taxa. Temperature shifts may alter the biochemical process (enzyme activities) within insects, and consequently deteriorate fertility, feeding patterns, survival rates, population dynamics, and dispersal patterns, and accordingly, change and modify the abundance, distribution, densities, and lifecycle of insects. The effect of climate change on insect ecology may disrupt pollination and hence, food security [15] and crop yield [16], because crop production depends mainly on climate variables and insect pests.

A similar study conducted at Rawdhat Khorium National Park (25°23’ N, and 47°17’ E), Northern Riyadh, Central Saudi Arabia, was conducted in order to determine the population of beetles in that area. The collection methods involved pitfall, UV-light, Malaise traps, net sweeping, beating vegetation, vacuuming, and hand-picking. The results of this study show that Tenebrionidae and Scarabeidae were the most abundant families [17].

Because of a lack of records in the KARR about the exact components of flora and fauna species, in addition to the needs for constructing base-line data, this study examined the insect occurrence and biodiversity in association with seasonal variation (spatio-temporal) during January–November 2023. The study was also interested in reflecting the presence and distribution of beneficial (pollinators, decomposers, and predators), and pest insects in order to assist in designing management plans within the study area. The study also constructs baseline data involving other categories such as non-insect arthropods, various classes of vertebrates, and plants.

## 2. Material and Methods

### 2.1. Study Area

The King Abdulaziz Royal Nature Reserve (KARR) covers an area of 28.345 km^2^, located between the coordinates E 45.19–46.57 and N 25.15–27.41. It encompasses parts of the Riyadh Province and the Eastern Province, two of the thirteen administrative Provinces of the Kingdom of Saudi Arabia (KSA). The reserve is divided into two parts: the southern and central regions, located in the northeastern area of Riyadh Province, cover a total area of 15.892 km^2^; meanwhile, the northern part of the reserve, known as As-Summan, situated in the northwestern part of the Eastern Province, covers an area of 12.436 km^2^. The central and southern areas of KARR, specifically Al-Tanahat, Al-Khafs, Noura, and the Al-Dahna Desert, are part of the eastern geological section of the Riyadh Province, known as the eastern portion of the Najd Plateau. The southern portion of KARR represents approximately 5.9% of the total area of the Arabian Shelf. The geological history of this region dates back to prehistoric eras. The study area is in a region characterized by a semi-arid to arid desert climate and the climatic factors (temperature, rainfall, relative humidity, and daylight) were obtained from the National Center for Meteorology KSA during the study period (Table 1).

Vegetation cover consists primarily of dwarf xeromorphic shrubs, annual plants, and perennial species. Sandy deserts dominate the landscape, interspersed with shrubs, shrublets, and some grasses.

**Figure 1:**
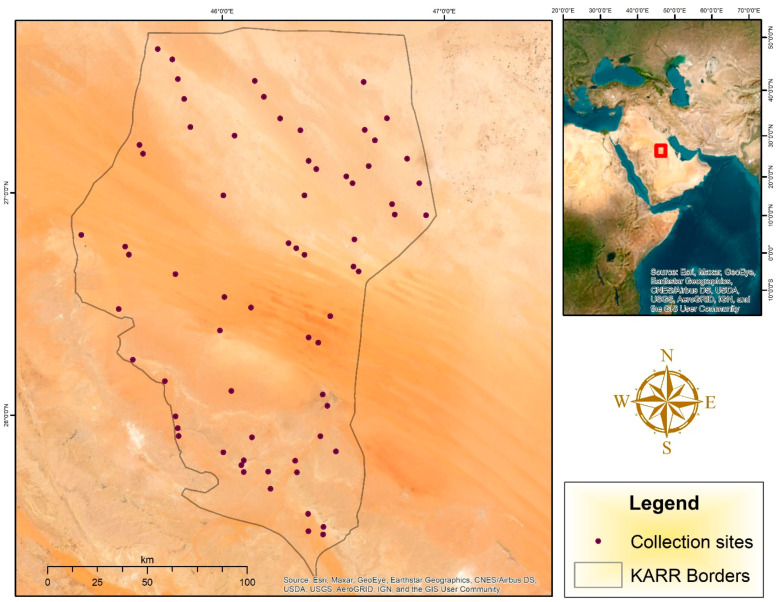
Map of the study area showing locations of different sites within KARR.

Sixty-eight locations were selected randomly within the KARR area to trap insects (Figure 1 and Table 2); the geographical position of each site was recorded using a Garmin eTrex 30 GPS (Lenexa Kansas City, USA) and Gaia program (https://help.gaiagps.com/hc/en-us (version number: Gaia Pro 2021) accessed on 24 January 2024) and all maps were prepared by ArcGIS Desktop 10.8.1 July 2020, Esri.

### 2.2. Sample Collection and Identification

This study employed active sampling methods, such as manual collection (mainly for Blattodea, Lepidoptera and Orthoptera) and night surveys (for moths and crickets). The study also adopted passive methods [18], which were useful for collecting nocturnal species and for the species that hide in response to the movement of the team members in the search area. Passive methods included the use of pitfall traps (mainly for ground beetles and ants), malaise traps (for Hymenoptera and Diptera), and blue vane traps (for bees and some wasps). The stick–paper method was also adopted for plant dwelling insects (Hemiptera and other plant pests, e.g., Coleoptera). One of each trap type was similarly and simultaneously put at each site in order to catch the maximum number of various insect species for constructing seasonal base-line data; a comparison of the effectiveness of each method was not conducted. Each trap was set up at 6:00 AM and collected after 24 h. The collected samples were transferred to labeled (number of site, date, and method of collection) bottles, half-filled with 70% ethanol, and then transported to the laboratory for immediate counting and identification.

Identification was first based on the key of Gillotts [19], which classified the obtained specimens to the order level. Then, the identification relied mainly on the morphological characteristics using the taxonomic keys for each insect order: Coleoptera [17,20,21,22], Hemiptera [20,23], Hymenoptera [20,24,25,26,27,28], Blattodea [29], Lepidoptera [20,30], and Orthoptera [20,31].

### 2.3. Statistical Analysis

Data collected were presented as relative abundance (%), density (number/site), distribution (positive sites), and the number of identified orders, families, species, and total collected specimens. Simpson’s diversity index (dominance index: D) was used to quantify the biodiversity of any given species in a community in a certain area (the higher the value, the higher the diversity or richness). Following the principals introduced by Simpson [32], which consider both the number of individual species and their relative abundance, the diversity index (D) for each species was calculated as follows:

$$D=\frac{ni(ni-1)}{N\left(N-1\right)}$$

The formula to calculate Simpson’s diversity (D) for insect communities was:

$$D=\frac{\sum ni\left(ni-1\right)}{N\left(N-1\right)}$$

where N = the total number of individual species in the population and n_i_ = the number of individual species.

The compliment (D) value ranges from 0 (indicating no distribution) to 1.0 (indicating distribution across all sites). This parameter was approved and used by KARR to quantify the diversity of animals within the reserved area. It also assisted the estimation of biodiversity decline [33].

The calculation of (1-D) is called Gini–Simpson index. Both Simpson’s diversity for the insect community and Gini–Simpson index (1-D) were not calculated as this study is concentrated on the occurrence and richness of species in respect to each season.

ANOVA (two factors) was used to determine type of difference between abundance of insect order members across different seasons at *p*-value (0.05) at both row (insect orders) and column (the seasons) levels.

## 3. Results

### 3.1. The Identification of the Collected Insects

**Figure 2:**
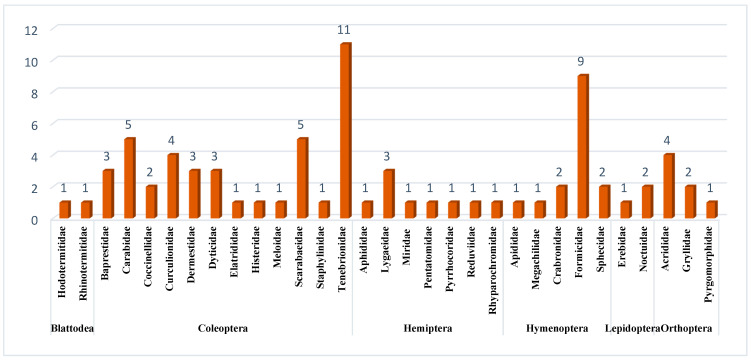
Biodiversity of insects within KARR according to No. of species within each family.

**Figure 3:**
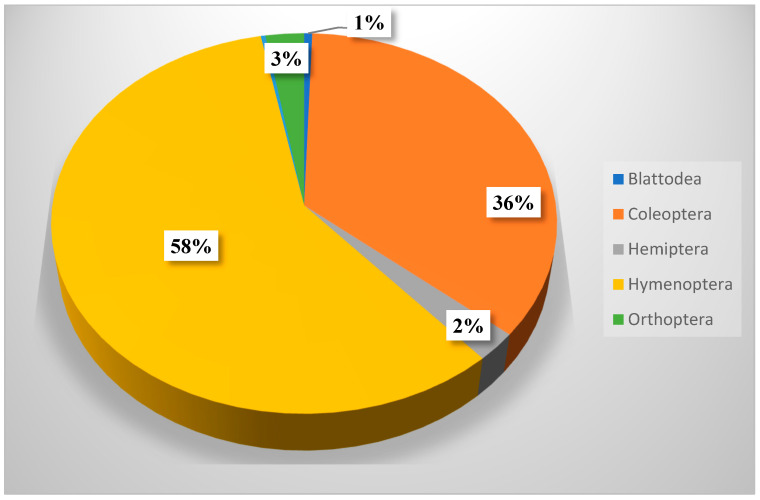
Relative abundance of insects within KARR according to No. of individual insects within each order (Lepidoptera represented less than 1%).

From 6320 trapped insects (excluding immature stages), the species belonged to six orders: Blattodea (36), Coleoptera (2264), Hemiptera (156), Hymenoptera (3672), Lepidoptera (21), and Orthoptera (171). Blattodea (the termites), were represented by two families and two species. Coleoptera contained 12 families and 38 species, 11 of them belonging to the family Tenebrionidae. Hemiptera contained seven families and nine species, three of them belonging to the family Lygaeidae. Hymenoptera contained 5 families and 15 species, 9 of them from Formicidae. Lepidoptera contained two families and three species. Orthoptera contained three families and seven species, four of them from the family Acrididae. The scientific names, author names, and years, in addition to the families, are presented in Table 3, while the biodiversity of species in respect to each order is presented in Figure 2. The relative abundance of each order is presented in Figure 3.

### 3.2. Biodiversity and Densities for the Winter Collection (January–March 2023)

**Figure 4:**
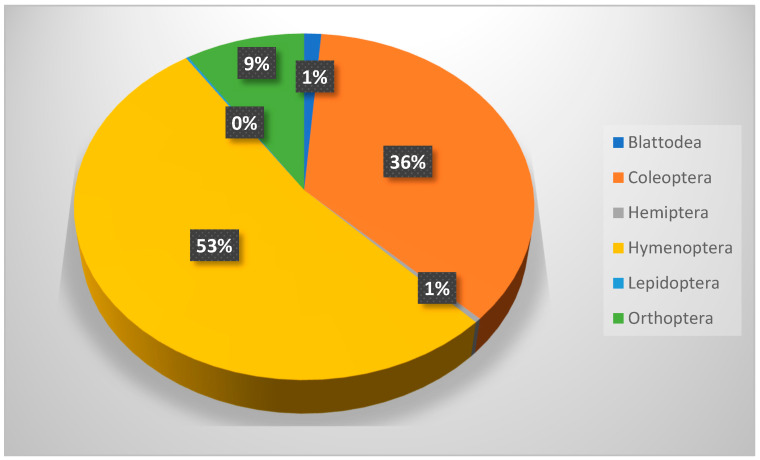
Relative abundance of insects trapped during winter season (January–March 2023). Lepidoptera represented less than 1%.

The winter collection (989 insects) involved six insect orders. In respect to the total number collected, Hymenoptera is the most distributed order (with one family, four species, and a count number of 523; D = 0.27), representing more than 50% of the total insects collected, followed by Coleoptera (7 families, 14 species, and total collection number of 326; D = 0.127). The collection also involved Orthoptera, Blattodea, and Lepidoptera (one family, one species for each), in addition to Hemiptera (two families and two species). The family and species names, corresponding to the number of positive sites (+ve site), the number collected, and the distribution index (D), are presented in Table 4, while relative abundances are presented in Figure 4. The immature stages of Coleoptera, Lepidoptera, and Hemiptera were noticed and recorded during this period. *B. polychresta* (D = 0.0033) and *M. angustata* (D = 0.0028) beetles, in addition to *Camponotus aegyptiacus* (D = 0.0368), *Messor meridionalis* (D = 0.0291), and *Cataglyphis viticoides* (D = 0.0221) ants, show a higher distribution index over other species collected during this season.

### 3.3. Biodiversity and Densities for the Spring Collection (May 2023)

**Figure 5:**
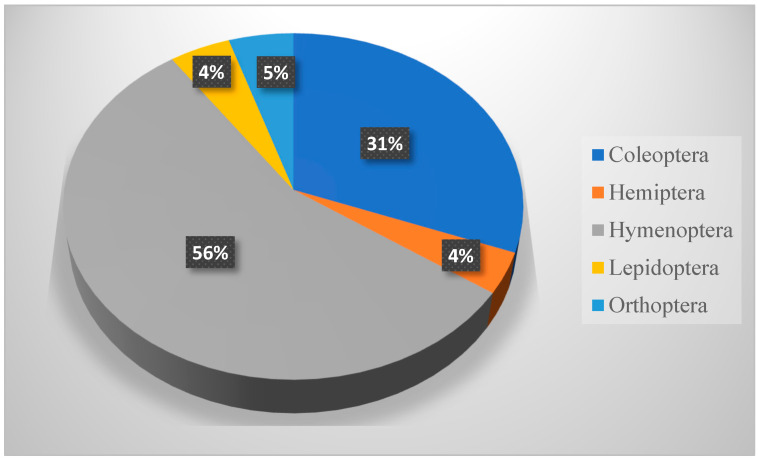
Relative abundance of insect orders trapped during spring season (May 2023).

The spring collection included 2275 insects belonging to 5 orders and 45 species, which is approximately double that of winter collection. Hymenoptera was the most abundant order (with two families, five species, and a count number of 1273; D = 0.313), which were highly distributed across KARR, followed by Coleoptera (10 families, 30 species, and a total collection number of 703; D = 0.095). The collection also involved three orders: Orthoptera (three families, three species, and total number of 114), Lepidoptera (two families, three species), in addition to Hemiptera (four families and four species; Table 5 and Figure 5). It was also noticed that the immature stages of Hemiptera, Orthoptera, and Lepidoptera were also recorded. *Camponotus aegyptiacus* (D = 0.086) ant, *B. polychresta* (D = 0.0019) beetle, in addition to Lepidoptera’s larvae (D = 0.0016), show a relatively higher distribution index over other species collected during spring season.

### 3.4. Biodiversity and Densities for the Summer Collection (August–September 2023)

**Figure 6:**
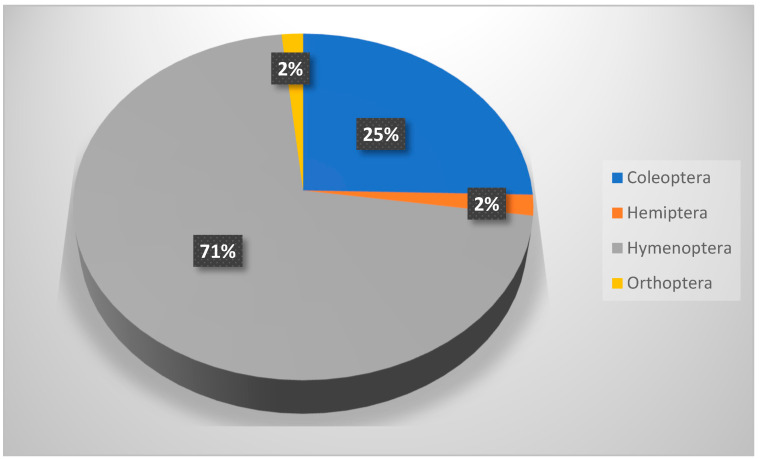
Relative abundance of insect order trapped during summer (August–September 2023).

The summer collection involved 1462 insects belonging to 4 orders and 30 species; most of them were darkling beetles and ants. Hymenoptera was the most abundant order (with 4 families, 11 species, and 1035 individuals; D = 0.501), followed by Coleoptera (6 families, 12 species, and 375 collected members; D = 0.065). The collection also involved two orders: Orthoptera (3 families, 3 species and total number of 24), and Hemiptera (2 families, 3 species, and total number of 28). *Camponotus aegyptiacus* (D = 0.043), *Camponotus foraminosus* (D = 0.036) ants, and *B. polychresta* (D = 0.0025) beetle, show a relatively higher distribution index over other species collected during summer season (Table 6 and Figure 6).

### 3.5. Biodiversity and Densities for the Autumn Collection (October–November 2023)

**Figure 7:**
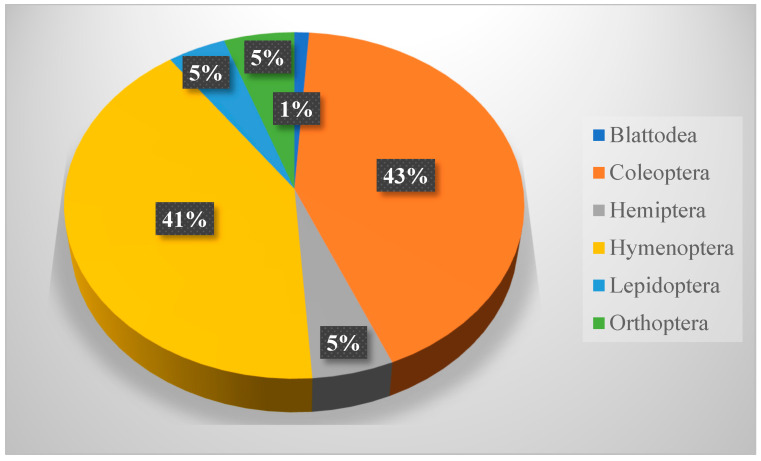
Relative abundance of insect orders trapped during autumn (October–November 2023).

The autumn collection involved 2042 insects belonging to 6 orders, 23 families, and 40 species, which is less than that of spring collection. Coleoptera was the most abundant order (8 families, 17 species, and 868 individuals; D = 0.180), followed by Hymenoptera (with 4 families, 10 species, and the a count number of 841; D = 0.169). The collection also involved Orthoptera (3 families, 5 species, and a total number of 111), Hemiptera (4 families, 5 species, and total number of 107), Lepidoptera (2 families, 3 species, and a total number of 92), in addition to Blattodea (2 families and 2 species, and a total number of 23). It was also noticed that the immature stages of all orders, except Hymenoptera and Blattodea, were also recorded. The *B. polychresta* (D = 0.0133) beetle, in addition to *Camponotus wroughtonii* (D = 0.0159) and *Messor meridioralis* (D = 0.0128) ants, show a relatively higher distribution index over other species collected during autumn season (Table 7 and Figure 7).

Table 8 summarizes the distribution of different insects across the seasons. It was clear that Blattodea was not recorded during summer and spring seasons, while Lepidoptera was not recorded during summer and only one individual was recorded during the winter season. Blattodea represented 0.57% of the total insect collected during this study, while Coleoptera represented 35.8%, Hemiptera represented 2.47%, Lepidoptera represented 0.33% (although considerable numbers of larvae were recorded but not identified as species), and Orthoptera represented 2.7%. Hymenoptera represented about 58% of all insects identified within KARR during the study period. It was also noticed that the spring collection (2059) was relatively greater than autumn (1842), summer (1462), and winter (957) collections. It was also noticed that there is no consistency for the abundance of insect species in respect to seasons, i.e., while some were flourished during summer or spring (Hymenoptera), others flourished during winter (Orthoptera) or autumn (Blattodea, Coleoptera, and Hemiptera).

ANOVA analysis shows a significant difference (at *p*-value 0.05) in the rows level (insect orders) but not in the columns level (seasons), i.e., the identified insects (orders) differ in their numbers and their richness across KARR, but did not differ significantly in their distribution among seasons.

## 4. Discussion

The aim of this study was to evaluate the biodiversity of insects (in terms of density and abundance) within the designated study area in addition to reflecting the abundance of some key species. Similar studies has been conducted in the Kingdom of Saudi Arabia in the years 2017 [17], 2018 [34], 2020 [35], 2021 [36], 2022 [37], and 2023 [38]. All these studies agreed that Coleoptera and Hymenoptera are the most abundant insects in the KSA.

Similar studies were conducted in some Arabic countries, e.g., the UAE, during 2014 [39] and 2023 [40], and Algeria [41] and they share similar results with the KSA regarding the flourishing of darkling beetles and some ant species. This finding may be attributed to the similarity of arid habitat in the above-mentioned countries.

Table 8 shows that members of Hymenoptera (about 58% of all collection) were the most dominant insect throughout this study. It also shows a significant difference between insect orders in their occurrence, but not in their distribution across seasons. Hymenopteran insects (ants, bees, and wasps) played important roles as pollinators, parasites, honey makers, and carnivores [36].

It was noticed that the immature stages of all orders, except Hymenoptera and Blattodea, were recorded during autumn, winter, and spring, but not during the summer season and this may be attributed to the shortage of food source and plant cover, while the temperature was higher and humidity was lower, as shown in Table 1. Biodiversity in KSA is mainly influenced by the arid climate, vast desert, extensive mountain ranges, and the long coastlines along the Red Sea and the Arabian Gulf [35]. Global climate change has affected abundance by altering various abiotic and biotic factors which in turn impacts species’ physiology, morphology, and life pattern. As such, systematic monitoring, assessment, and conservation are necessary to protect biodiversity in Saudi Arabia’s aquatic and terrestrial ecosystems, in line with Vision 2030 [12].

In respect to microhabitat, few species of insects are restricted to certain plants. One of these insects is *Steraspis speciosa* (the xylophagus beetle) which was recorded in this study. It is a potential cause of significant damage and death to *Acacia* trees in Saudi Arabia in the driest environments. This beetle is considered a pest of *Acacia* tree [22], while *Hypolixus pica* beetles (recorded in this study) are considered as beneficial insects and a potential biological control agent against *Amaranthus* weeds [42].

Few species of beetles with specialized adaptations appeared in certain seasons. One of these species is the carrion beetle *Dermestes maculatus*, which was recorded at two sites (four individuals; 0.18% of all Coleoptera) during the spring season only. It is an example of a spring-flourishing insect. This beetle feeds on dead vertebrate bodies and decomposes the flesh [43], so it has been used for the preparation of vertebrate skeletons [44]. The abundance of this insect is very important to decompose dead vertebrates within the KARR.

Another example highlighting the same concern is the *Hydroglyphus signatellus* beetle (seven individuals; 0.31% of all Coleoptera), which appeared only during the autumn season, swimming in stagnant rainwater. This species is known as a predaceous diving beetle [45].

Five species belonging to the Scarabaeidea family were recorded during this study. These beetles feed exclusively on herbivore and omnivore feces, and hence, are named dung beetles. These beetles inhabit and are adapted to live in desert, savanna, and forest ecosystems, but do not like very cold or hot habitats. These beetles play important ecological roles, first by burying and consuming dung, which helps to improve soil structure and plant growth; second by helping to distribute seeds present in dung; and third by helping in environmental control by removing the dung of cattle and assisting animal husbandry against vectors and flies, saving millions of dollars spent in cattle industries [46].

Table 4, Table 5, Table 6 and Table 7 show that *Blaps polychresta* dominated across the study periods (466 individuals; 20.58% of all Coleoptera). *Blaps* is a genus of darkling beetle, represented in this study by two genera. It was trapped within 54 sites out of 68. Generally, there are more than 30 known species of *Blaps*, mainly distributed in Eurasia and Australia [21].

Table 2 shows that the family Tenebrionidae (the darkling beetles) has a high species richness across this study (represented by 12 species, 1668 individuals, and 73.67% of all Coleoptera), and this may be as a result of its special adaptations to live in arid and semiarid habitats. They prefer vegetated (shrub or grass) habitats. This finding can be attributed to the fact that vegetation tends to reduce the risk of desiccation and predation and offers better oviposition location; additionally, these beetles are detritivores [47]. The predatory darkling beetle can move easily between the vegetation to pursue prey. It is well known that the microorganisms which live in the digestive system of darkling beetles are used to decompose plant litter and recycle the nutrients into the soil to improve its nutrient composition, and hence, help in increasing plant production. Some darkling beetles (e.g., *Calosoma*) are more important predators than many other desert predators. The abundance of these beetles year-round in desert areas makes them an available source of food for reptiles, birds, and small mammals. It is very important to mention that none of the *Blap* species were recorded as pests [48].

Another member of Tenebrionidae beetle family that was widely distributed within the KARR is the beetle *Mesostena angustata* (recorded within 52 sites out of 68, with a total of 347 individuals (20.8% of all darkling beetles) trapped across the study period). In some reports, this beetle is well known as a predator and was noticed to feed on *Messor intermedius* ant [49]. In the current study, this beetle was noticed within some termite colonies, feeding on them. Beetles (specially Tenebrionidae) are widely distributed and dominate across several Provinces in the KSA [33,34,35,37], UAE [40], Algeria [41], Iran [49], Qatar [50], Iraq [51], Kuwait [52], and Israel [53], but not in India [54] where Lepidoptera is dominates over Coleoptera and Hymenoptera.

The *Pimelia* beetle was also recorded in this study with a remarkable abundance (227 individuals; 13.61% of all darkling beetles). It is an example of a good desert-adapted beetle. It survives in arid climates and desert environments (high temperature, low humidity, excessive radiant energy, low and irregular rainfall, long periods of drought, strong winds, and unstable sand substrates) in virtue of some adaptations, including it losing its ability to fly. Its morphological adaptations include the waxy layers of the epicuticle, the fused sclerites (that minimize water loss), the subelytral cavity (that allowed relative humidity to spiracles and reduce water loss), and the structure of the body surface (which reflects and scatters radiant energy). Diurnal activity early morning and late evening in addition to burrowing behavior helps a lot in heat regulation during hot days [53]. It was suggested that the abundance of darkling beetles can be used as an environmental bioindicator for pesticides [55].

Concerning the ecological adaptations, the darkling beetle, *Prionotheca coronata* (61 individuals distributed within 24 sites), has unique morphological and behavioral adaptations to the desert area. It possesses sharp spines along its abdominal margin and along its inner hind legs (for this reason it is named urchin beetle), used against vertebrate or invertebrate enemies [56].

Two individuals of *triatoma* (Hemiptera) were recorded in only one site within the KARR. *Triatoma* is a blood-sucking bug and can transmit Chagas disease that infects human and other mammals. Its saliva may cause allergic reactions to some people; hence, it was ranked with the medical and veterinary insects [57]. Although only two *triatoma* bugs were recorded, attention must be focused on their medical value to the local people and their domestic animals.

*Aphis nerii* was also reported in this study (73 individuals; 46.79% of all Hemiptera insects). It is considered to be a plant pest that causes serious damage to various crops due to its feeding habit, resulting in economic losses. Some Hemiptera insects can transmit various plant pathogens (e.g., viruses, bacteria, and phytoplasmas) [58]. Their successive feeding process in addition to their potential to transmit plant pathogens may hurt crops and cause a negative impact on agricultural production. The interactions between plants and Hemiptera are multi-faceted; part of this interaction is related to the plant biosynthesis of some molecules and the adaptation of some defense mechanisms. Insects struggle to obtain nutrients from plants and to assure shelter and oviposition place within plants. The plant then responds to insect infestation by developing physical barriers to prevent insect gaining access to plants and by producing anti-nutrition (deterrent) compounds. In turn, insects adapt to avoid plants’ defense strategies. The development of competing strategies that benefit both plants and insects will never stop, as it is a concept of survival [59,60].

Two species of termites were recorded in this study, but there are 30 termite species distributed in the Arabian Peninsula, belonging to 4 families and 9 genera, of which 27 species are known in Saudi Arabia (three of them are previously recorded in Riyadh Province: *Anacathotermes ochraceous, Psammotermes hypostoma* and *Coptotermes heimi*) [29]. *Anacathotermes* and *Psammotermes* termites are distributed in the desert and semi-desert areas of North Africa, Middle East, and Southwest Asia [61].

Although termites are considered as wood pests in forests, they are also considered as main decomposers in arid and semi-arid habitats. Termites also act as soil engineers, since they improve the physical (texture) and chemical properties of the soil through acting as bioturbators and as weathering agents of clay minerals (e.g., iron and calcium) [62,63].

Table 2 shows the relatively high abundance of ant species: *Camponotus* (2233 individuals, 60.81% of all Hymenoptera and 35.22% of all collected insects), *Messor* (817 individuals, 22.25% of all Hymenoptera and 12.88% of all collected insects), and *Cataglyphis* (599 individuals, 16.38% of all Hymenoptera and 9.45% of all collected insects). These species are reported within some Provinces of the KSA [36]. Ants played an important ecological and agricultural role in seedling establishment and plant composition, specifically in the arid habitat [64]. Ants are also considered to be cheap, clean, and accurate bio-indicator tools for diagnosing soil fertility. The physical properties and chemical contents (e.g., nitrogen and phosphorus) of soil are significantly improved within three weeks of ant presence [65]. Concerning the distribution of ants, they are widely found in agricultural land, dry land, vegetative land, and human houses, without any significant differences between these habitats [66]. The distribution of ants across a trees is correlated with tree trunk size, topography, and tree species [67].

*Schistocerca. gregaria* (the desert locust), which was recorded at five sites in the KARR, is a highly voracious and polyphagous insect, and is considered to be one of the most dangerous and destructive migratory pests in the world against various crops. It can travel hundreds of kilometers a day; thus, its population size cannot be easily estimated. Integrated control methods, in addition to monitoring techniques, should be adopted against this serious pest [68,69] and in the KARR.

Few members of bees, wasps, and Lepidoptera’s were recorded in this study. Bees (honey bees and megachilids), Lepidoptera (butterfly and moth), and aculeate wasps, are the main pollinators. Flowering plants in turn encourage these insects by providing nectar and high-protein foods in the form of pollen grains. Their adhesive and hairy legs or bodies, in addition to the presence of some basket-like structures in some insect species, assists in pollination. It was noticed that insecticide application against some plant pests occasionally hurt these pollinators (as non-target organisms) and the consequences will be reduction in pollinator activity and a collapse in plant production [70,71].

The variation in species composition in any ecosystem is dependent on its assemblage, dynamics, inter and intra-specific interactions, etc., which changes among different microhabitats [72], which is very obvious in the biodiversity of insect species reported in this study in respect to the variation in habitats across different sites and seasons within the KARR. No specific patterns or features are noticed during this study to indicate climate change since the same species with the same morphological features are distributed within the KSA and neighboring countries. The occurrence and distribution of various pollinators, seed distributors, decomposers, predators, plant pests, and medical and veterinary insects, in addition to considerable numbers of immature stages and other mentioned insects across the seasons within the KARR, in addition to other neighboring provinces and countries, reflects the status of the ecological system and its capacity to host this diversity. It is also worth noting that none of the mutation signs or features were noticed during the identification process.

The integration of the obtained data with that of other parallel survey studies (non-insect arthropods, reptiles, birds, and mammals), will help the KARR in planning suitable conservation plans. The data of the further surveys will help to determine whether the occurrence line of insects is stable, increases, or declines.

## 5. Conclusions

Insect density and biodiversity were observed to be relatively lower during the winter season (January–March) and autumn (October–November), but relatively higher during spring (May) and summer (August–September). The number of identified insects in each order differ significantly, but their abundance during the seasons are do not differ substantially, i.e., no specific season provides the optimum conditions for all identified insects to flourish. The immature stages of all orders, except Hymenoptera and Blattodea, were not recorded during summer season. This study also showed the presence of some plant pests, disease vectors, pollinators, decomposers, and potential bio-control insects distributed within the study area.

## Figures and Tables

**Table 1:** Weather (means) in KARR according to the National Center for Meteorology KSA (2023).

Factors (Means)	Winter	Spring	Summer	Autumn
**Temperature** (°C)	15	35	40	30
**Relative Humidity (%)**	37%	24%	8%	55%
**Daylight (h/minute)**	10:52	12:39	13:25	11:58
**Rainfall** (mm/season)	40	60	0	25

**Table 2:** Localities and coordinates of the study area during the study.

Point	Latitude	Longitude	Point	Latitude	Longitude
1	27.64687	45.70956	35	26.44472	46.48722
2	27.60016	45.77615	36	26.72098	46.37087
3	27.49881	46.637	37	26.75791	45.56311
4	27.50362	46.14716	38	26.7219	45.57994
5	27.25699	46.05608	39	26.63353	45.78956
6	27.5113	45.80098	40	26.53097	46.01017
7	27.42217	45.82912	41	26.47676	45.5344
8	27.43199	46.18824	42	26.48346	46.13026
9	27.29568	45.85748	43	26.32537	46.43316
10	27.33444	46.2617	44	26.34874	46.38948
11	27.3353	46.74194	45	26.38056	45.98999
12	27.23572	46.68774	46	26.09198	46.45269
13	27.28289	46.64212	47	26.15203	45.7425
14	27.2811	46.35249	48	26.04162	46.47419
15	27.15268	46.83274	49	26.10807	46.04133
16	27.14344	46.38964	50	25.9934	45.79071
17	27.04274	46.88768	51	25.90428	46.44288
18	27.12033	46.65985	52	25.79416	46.32906
19	27.07357	46.55927	53	25.8992	46.13403
20	27.10671	46.42414	54	25.90502	45.80391
21	26.94894	46.76561	55	25.74185	46.33688
22	27.04254	46.58772	56	25.79536	46.09838
23	26.98761	46.37087	57	25.83223	46.00561
24	27.21515	45.62814	58	25.94043	45.8005
25	26.98761	46.00561	59	25.74518	46.20714
26	26.89873	46.91875	60	25.74335	46.09692
27	26.9016	46.77738	61	25.77428	46.08666
28	26.7727	46.29943	62	25.83619	46.51288
29	27.17566	45.6439	63	25.4964	46.45628
30	26.79004	46.59523	64	25.55459	46.38676
31	26.66731	46.59169	65	25.46241	46.45496
32	26.75088	46.33357	66	25.47711	46.3885
33	26.80986	45.36641	67	25.66739	46.21809
34	26.64558	46.61481	68	26.24909	45.59824

**Table 3:** Specification of the identified insects within KARR during study period.

Order	Family	Scientific Name	(+ve) Sites	Total No.
Blattodea	Hodotermitidae	*Anacanthotermes ochraceous* (Burmeister, 1839)	6	29
	Rhinotermitidae	*Psammotermes hybostoma* Desneux, 1902	2	7
Coleoptera	Baprestidae	*Steraspis speciosa* (Klug, 1829)	8	19
		*Julodis euphratica* Laporte & Gory, 1835	2	5
		*Julodis* sp. Eschscholtz, 1829	1	1
	Carabidae	*Amara aulica* (Panzer, 1797)	11	68
		*Anthia duodecimguttata* Bonelli, 1813	31	214
		*Brachinus nobilis* Dejean, 1831	4	33
		*Calosoma imbricatum* Klug, 1832	22	68
		*Scarites procerus* Fischer von Waldheim, 1828	1	1
	Coccinellidae	*Coccinella undecimpunctata* Mulsant, 1850	4	13
		*Diomus rubidus* (Motschulsky, 1837)	2	3
	Curculionidae	*Hypera brunnipennis* (Boheman, 1834)	4	5
		*Hypolixus pica* (Fabricius, 1798)	2	6
		*Mecinus longulus* (Desbrochers des Loges, 1893)	1	2
		*Pycnodactylopsis tomentosa* (Fåhraeus, 1842)	1	1
	Dermestidae	*Attagenus fasciolatus* (Solsky, 1876)	1	2
		*Attagenus lobatus* Rosenhauer, 1856	3	4
		*Dermestes maculatus* De Geer, 1774	2	4
	Dyticidae	*Hydroglyphus signatellus* (Klug, 1834)	3	7
	Elatrididae	*Lacon modestus* (Boisduval, 1835)	10	25
	Histeridae	*Teretrius pulex* Fairmaire, 1877	2	3
	Meloidae	*Mylabris elegans* Olivier, 1811	4	5
	Scarabaeidae	*Aphodius arabicus* Harold, 1875	3	6
		*Aphodius lividus* (Olivier, 1789)	3	4
		*Maladera insanabilis* (Brenske 1894)	4	23
		*Podalgus cuniculus arabicus* (Fairmaire, 1895)	1	1
		*Rhyssemus saoudi* Pittino, 1984	20	72
	Staphylinidae	*Philonothus* sp. Stephens, 1829	1	1
	Tenebrionidae	*Adesmia cancellata* (Klug, 1830)	51	279
		*Akis elevata* Solier, 1836	39	180
		*Blaps kollari* Seidlitz, 1893	3	8
		*Blaps polychresta* (Forskål, 1775)	54	466
		*Gonocephalum prolixum* (Erichson, 1843)	1	6
		*Mesostena angustata* (Fabricius, 1775)	52	347
		*Pimelia arabica* (Klug, 1830)	49	175
		*Pimelia* sp. Fabricius, 1775	25	52
		*Prionotheca coronata ovalis* Ancey, 1881	24	61
		*Trachyderma philistina* Reiche & Saulcy, 1857	20	88
		*Zophosis punctata* Brullé, 1832	3	6
Hemiptera	Aphididae	*Aphis nerii* Fonscolombe, 1841	9	73
	Lygaeidae	*Dieuches armipes* (Fabricius, 1794)	5	13
		*Lethaeus fulvovarius* Puton, 1884	2	2
		*Spilostethus pandurus* (Scopoli, 1763)	8	31
	Miridae	*Tylorilygus apicalis* (Fieber, 1861)	1	1
	Pentatomidae	*Phyllocephala negus* (Distant, 1900)	4	5
	Pyrrhocoridae	*Scantius aegypius* (Linnaeus, 1758)	7	20
	Reduviidae	*Triatoma* sp. (Laporte, 1832)	1	2
	Rhyparochromidae	*Beosus maritimus* (Scopoli, 1763)	3	9
Hymenoptera	Apididae	*Apis mellifera* (Linnaeus, 1758)	4	5
	Megachilidae	*Megachile* sp. Latreille, 1802	1	4
	Crabronidae	*Bembix* sp. Fabricius, 1775	3	4
		*Mescophus* sp. Jarine, 1807	2	4
	Formicidae	*Camponotus aegyptiacus* Emery, 1915	44	1297
		*Camponotus foraminosus* Forel, 1879	18	394
		*Camponotus* sp. Mayr, 1861	7	91
		*Camponotus wroughtonii* Forel, 1893	23	451
		*Cataglyphis bicolor* (Fabricius, 1793)	6	32
		*Cataglyphis* sp. Forster, 1850	32	281
		*Cataglyphis viticoides* (Andre, 1881)	22	286
		*Messor alalocapius* (Ruzsky, 1902)	27	172
		*Messor meridionalis* (Andre, 1883)	14	645
	Sphecidae	*Ammophila* sp. W. Kirby, 1798	1	2
		*Sphex pruinosus* Germar, 1817	3	4
Lepidoptera	Erebidae	*Eublemma pallidula* (Herrich-Schaffer, 1856)	5	8
	Noctuidae	*Heliothis peltigera* (Denis & Schiffermüller, 1775)	6	7
		*Spodoptera mauritia* (Boisduval, 1833)	4	6
Orthoptera	Acrididae	*Cyrtacanthacris tatarica* (Linnaeus, 1758)	1	1
		*Schistocerca gregaria* (Forsskål, 1775)	5	22
		*Rtuxalis nasuta* (Linnaeus, 1758)	1	1
		*Scintharista notabilis* (Walker, 1870)	5	14
	Gryllidae	*Gryllodes sigillatus* (Walker, 1869)	6	101
		*Gryllus bimaculatus* De Geer, 1773	7	30
	Pyrgomorphidae	*Pyrogomorpha conica* (Olivier, 1791)	2	2
Total				**6320**

**Table 4:** Insect species trapped during winter season (January–March 2023).

Order	Family	Species	(+ve) Sites	No.	D
Blattodea	Hodotermitidae	*Anacanthotermes ochraceous*	4	13	1.56 × 10^−4^
Coleoptera	Baprestidae	*Steraspis speciosa*	7	15	2.11 × 10^−4^
	Carabidae	Carabidae larvae	7	30	8.73 × 10^−4^
		*Amara aulica*	8	16	2.41 × 10^−4^
		*Anthia duodecimguttata*	2	13	1.56 × 10^−4^
		*Brachinus nobilis*	2	17	2.73 × 10^−4^
		*Calosoma imbricatum*	7	13	1.56 × 10^−4^
	Coccinellidae	*Diomus rubidus*	2	3	6.14 × 10^−6^
	Dermestidae	*Attagenus fasciolatus*	1	2	2.04 × 10^−6^
	Meloidae	*Mylabris elegans*	3	4	1.23 × 10^−5^
	Scarabaeidae	*Rhyssemus saoudi*	6	27	7.04 × 10^−4^
	Tenebrionidae	*Adesmia cancellata*	24	38	0.0014
		*Akis elevate*	5	15	2.11 × 10^−4^
		*Blaps polychresta*	32	58	0.0033
		*Mesostena angustata*	33	53	0.0028
		*Pimelia* sp.	25	52	0.0026
				**356**	**0.1267**
Hemiptera	Lygaeidae	*Spilostethus pandurus*	1	2	2.04 × 10^−6^
	Pentatomidae	*Phyllocephala negus*	1	1	0.00
		Nymph	1	1	0.00
				**4**	**1.23 × 10^−5^**
Hymenoptera	Formicidae	*Camponotus aegyptiacus*	7	192	0.0368
		*Camponotus foraminosus*	3	11	1.1 × 10^−4^
		*Cataglyphis viticoides*	14	149	0.0221
		*Messor meridionalis*	1	171	0.0291
				**523**	**0.2738**
Lepidoptera	Noctuidae	*Heliothis peltigera*	1	1	0.00
		Larvae	1	1	0.00
				**2**	**2.04 × 10^−6^**
Orthoptera	Gryllidae	*Gryllodes sigillatus*	3	91	0.0082
				**989**	

**Table 5:** Insect species trapped during spring season (May 2023).

Order	Family	Species	(+ve) Sites	No.	D
Coleoptera	Buprestidae	*Steraspis speciosa*	2	4	2.32 × 10^−6^
		*Julodis euphratica*	2	5	3.87 × 10^−6^
		*Julodis* sp.	1	1	0.00
	Carabidae	*Amara aulica*	3	48	4.36 × 10^−4^
		*Anthia duodecimguttata*	14	87	0.0014
		*Brachinus nobilis*	2	16	4.64 × 10^−5^
		*Calosoma imbricatum*	19	55	5.74 × 10^−4^
		*Scarites procerus*	1	1	0.00
	Curculionidae	*Hypera brunnipennis*	1	2	3.86 × 10^−7^
		*Hypolixus pica*	2	6	5.80 × 10^−6^
		*Mecinus longulus*	1	2	3.86 × 10^−7^
	Dermestidae	*Attagenus lobatus*	2	3	1.16 × 10^−6^
		*Dermestes maculatus*	2	4	2.32 × 10^−6^
	Elateridae	*Lacon modestus*	2	3	1.16 × 10^−6^
	Histeridae	*Teretrius pulex*	2	3	1.16 × 10^−6^
	Meloidae	*Mylabris elegans*	1	1	0.00
	Scarabaeidae	*Aphodius arabicus*	1	3	1.16 × 10^−6^
		*Maladera insanabilis*	2	5	3.87 × 10^−6^
		*Podalgus cuniculus*	1	1	0.00
		*Rhyssemus saoudi*	13	25	1.15 × 10^−4^
	Staphylinidae	*Philonothus* sp.	1	1	0.00
	Tenebrionidae	*Adesmia cancellata*	19	83	0.0013
		*Akis elevate*	14	57	6.17 × 10^−4^
		*Blaps kollari*	3	8	1.08 × 10^−5^
		*Blaps polychresta*	40	99	0.0019
		*Gonocephalum prolixum*	1	6	5.80 × 10^−6^
		*Mesostena angustata*	29	90	0.0015
		*Pimelia arabica*	19	54	5.53 × 10^−4^
		*Prionotheca coronata*	8	24	1.06 × 10^−4^
		*Zophosis punctate*	3	6	5.80 × 10^−6^
				**703**	**0.0954**
Himeptera	Lygaeidae	Nymph	6	53	5.32 × 10^−4^
		*Lethaeus fulvovarius*	2	2	3.86 × 10^−7^
	Miridae	*Tylorilygus apicalis*	1	1	0.00
	Pyrrhocoridae	*Scantius aegypius*	7	20	7.34 × 10^−5^
	Reduviidae	*Triatoma* sp.	1	2	3.86 × 10^−7^
				**78**	**0.00116**
Hymenoptera	Apididae	*Apis mellifera*	3	4	2.32 × 10^−6^
	Formicidae	*Camponotus aegyptiacus*	44	669	0.0864
		*Cataglyphis* sp.	32	281	0.0152
		*Cataglyphis viticoides*	9	89	0.0015
		*Messor meridionalis*	12	230	0.0102
				**1273**	**0.313**
Lepidoptera		Larvae	9	91	0.0016
	Erebidae	*Eublemma pallidula*	4	7	8.12 × 10^−6^
	Noctuidae	*Heliothis peltigera*	3	4	2.32 × 10^−6^
		*Spodoptera mauritia*	3	5	3.87 × 10^−6^
				**107**	**0.00219**
Orthoptera	Acrididae	Nymph	2	5	3.87 × 10^−6^
		*Scintharista notabilis*	2	4	2.32 × 10^−6^
	Gryllidae	Nymph	8	67	8.55 × 10^−4^
		*Gryllodes sigillatus*	3	8	1.08 × 10^−5^
		*Gryllus bimaculatus*	7	30	1.68 × 10^−4^
				**114**	**0.00249**
Total				**2275**	

**Table 6:** Insect species trapped during summer season (August–September 2023).

Order	Family	Species	(+ve) Sites	No.	D
Coleoptera	Carabidae	*Anthia duodecimguttata*	25	51	0.0012
	Coccinellidae	*Coccinella undecimpunctata*	2	8	2.62 × 10^−6^
	Curculionidae	*Pycnodactylopsis tomentosa*	1	1	0.00
	Elateridae	*Lacon modestus*	2	1	0.00
	Scarabaeidae	*Maladera insanabilis*	2	18	1.43 × 10^−4^
	Tenebrionidae	*Adesmia cancellata*	23	61	0.0017
		*Akis elevata*	19	46	9.69 × 10^−4^
		*Blaps polychresta*	31	73	0.0025
		*Mesostena angustata*	32	65	0.0019
		*Pimelia arabica*	14	19	1.60 × 10^−4^
		*Prionotheca coronate*	16	17	1.27 × 10^−4^
		*Trachyderma philistina*	6	10	4.21 × 10^−5^
				**375**	**0.0657**
Hemiptera	Lygaeidae	*Dieuches armipes*	4	12	6.18 × 10^−5^
		*Spilostethus pandurus*	1	13	7.30 × 10^−5^
	Pentatomidae	*Phyllocephala negus*	2	3	2.81 × 10^−6^
				**28**	**3.54 × 10^−4^**
Hymenoptera	Apididae	*Apis mellifera*	1	1	0.00
	Crabronidae	*Bembix* sp.	3	4	5.62 × 10^−6^
	Formicidae	*Camponotus aegyptiacus*	36	306	0.0437
		*Camponotus foraminosus*	18	281	0.0368
		*Camponotus* sp.	1	8	2.62 × 10^−5^
		*Camponotus wroughtonii*	14	193	0.0173
		*Cataglyphis bicolor*	2	9	3.37 × 10^−5^
		*Cataglyphis viticoides*	5	48	0.0011
		*Messor alalocapius*	1	172	0.0138
		*Messor meridioralis*	1	12	6.18 × 10^−5^
	Sphecidae	*Sphex pruinosus*	1	1	0.00
				**1035**	**0.5010**
Orthoptera	Acrididae	*Schistocerca gregaria*	4	20	1.78 × 10^−4^
		*Scintharista notabilis*	1	2	9.36 × 10^−7^
	Gryllidae	*Gryllodes sigillatus*	1	2	9.36 × 10^−7^
				**24**	**2.58 × 10^−4^**
Total				**1462**	

**Table 7:** Insect species trapped during autumn season (October–November 2023).

Order	Family	Species	(+ve) Sites	No.	D
Blattodea	Hodotermitidae	*Anacanthotermes ochraceus*	3	16	5.75 × 10^−5^
	Rhinotermitidae	*Psammotermes hybostoma*	2	7	1.01 × 10^−5^
				**23**	**0.00012**
Coleoptera	Carabidae	*Amara aulica*	2	4	2.88 × 10^−6^
		*Anthia duodecimguttata*	15	63	0.00093
	Coccinellidae	*Coccinella undecimpunctata*	2	5	4.79 × 10^−6^
		*Coccinella* larvae	1	6	7.19 × 10^−6^
	Curculionidae	*Hypera brunnipennis*	3	3	1.44 × 10^−6^
	Dermestidae	*Attagenus lobatus*	1	1	0.00
	Dyticidae	*Hydroglyphus signatellus*	3	7	1.01 × 10^−5^
	Elatrididae	*Lacon modestus*	7	21	0.00010
	Scarabaeidae	*Aphodius arabicus*	2	3	1.44 × 10^−6^
		*Aphodius lividus*	3	4	2.88 × 10^−6^
		*Rhyssemus saoudi*	4	20	9.11 × 10^−5^
	Tenebrionidae	*Adesmia cancellata*	43	87	0.00179
		*Akis elevate*	22	62	0.000907
		*Blaps polychresta*	51	236	0.0133
		*Mesostena angustata*	40	139	0.00460
		*Pimelia arabica*	36	102	0.00247
		*Prionotheca coronate*	8	20	9.11 × 10^−5^
		*Trachyderma philistina*	15	78	0.00144
		Larvae	2	8	1.34 × 10^−5^
				**868**	**0.180**
Hemiptera	Aphididae	*Aphis nerii*	9	73	0.00126
	Lygaeidae	Nymph	2	2	4.78 × 10^−7^
		*Dieuches armipes*	1	1	0.00
		*Spilostethus pandurus*	6	16	5.75 × 10^−5^
	Pentatomidae	*Phyllocephala negus*	1	1	0.00
		Nymph	3	5	4.80 × 10^−6^
	Rhyparochromidae	*Beosus maritimus*	3	9	1.721 × 10^−5^
				**107**	**0.00272**
Hymenoptera	Megachilidae	*Megachile* sp.	1	4	2.88 × 10^−6^
	Crabronidae	*Mescophus* sp.	2	4	2.88 × 10^−6^
	Formicidae	*Camponotus aegyptiacus*	15	130	0.00402
		*Camponotus foraminosus*	6	102	0.00247
		*Camponotus* sp.	6	83	0.00163
		*Camponotus wroughtonii*	17	258	0.0159
		*Cataglyphis bicolor*	5	23	0.000121
		*Messor meridioralis*	27	232	0.0128
	Sphecidae	*Ammophila* sp.	1	2	4.78 × 10^−7^
		*Sphex pruinosus*	2	3	1.44 × 10^−6^
				**841**	**0.169**
Lepidoptera	Erebidae	*Eublemma pallidula*	1	1	0.00
	Noctuidae	*Heliothis peltigera*	2	2	4.78 × 10^−7^
		*Spodoptera mauritia*	1	1	0.00
		Larvae	23	88	0.00183
				**92**	**0.0020**
Orthoptera	Acrididae	*Cyrtacanthacris tatarica*	1	1	0.00
		*Schistocerca gregaria*	1	2	4.78 × 10^−7^
		*Rtuxalis nasuta*	1	1	0.00
		*Scintharista notabilis*	2	8	1.34 × 10^−5^
		Nymph	30	73	0.00126
	Gryllidae	Nymph	14	24	0.000132
	Pyrgomorphidae	*Pyrogomorpha conica*	2	2	4.78 × 10^−7^
				**203**	**0.00983**
Total				**2042**	

**Table 8:** Summary of the distribution of order members during different seasons.

Order	Summer	Autumn	Winter	Spring	Total (%)
Blattodea	0	23	13	0	**36 (0.57%)**
Coleoptera	375	860	326	703	**2264 (35.8%)**
Hemiptera	28	100	3	25	**156 (2.47%)**
Hymenoptera	1035	841	523	1273	**3672 (58.1%)**
Lepidoptera	0	4	1	16	**21 (0.33%)**
Orthoptera	24	14	91	42	**171 (2.7%)**
**Total**	**1462**	**1842**	**957**	**2059**	**6320**
ANOVA					
Rows	*p*-value (1.59 × 10^−6^) < 0.05				
Columns	*p*-value (0.24) > 0.05				

mmature stages are not included in this table.

## Data Availability

All data analyzed are included within the article.
